# Experiences of Health Care Access Challenges for Back Pain Care Across the Rural-Urban Continuum in Canada: Protocol for Cross-sectional Research

**DOI:** 10.2196/42484

**Published:** 2022-12-19

**Authors:** Katie Crockett, Stacey Lovo, Alison Irvine, Catherine Trask, Sarah Oosman, Veronica McKinney, Terrence McDonald, Nazmi Sari, Bertha Carnegie, Marie Custer, Stacey McIntosh, Brenna Bath

**Affiliations:** 1 School of Rehabilitation Science University of Saskatchewan Saskatoon, SK Canada; 2 Department of Biomedical Engineering and Health Systems (Medicinteknik och Hälsosystem), School of Engineering Sciences in Chemistry, Biotechnology, & Health (Kemi, Bioteknologi och Hälsa) Kungliga Tekniska Högskolan Royal Institute of Technology Stockholm Sweden; 3 Canadian Centre for Health and Safety in Agriculture University of Saskatchewan Saskatoon, SK Canada; 4 College of Medicine University of Saskatchewan Saskatoon, SK Canada; 5 Departments of Family Medicine and Community Health Sciences University of Calgary Calgary, AB Canada; 6 Department of Economics University of Saskatchewan Saskatoon, SK Canada; 7 Patient Partner University of Saskatchewan Saskatoon, SK Canada

**Keywords:** low back pain, rural health, rehabilitation, health services

## Abstract

**Background:**

Back pain is common and costly, with negative impacts on both individuals and the health care system. Rural, remote, and Indigenous populations are at greater risk of experiencing back pain compared to urban and non-Indigenous populations. Potential barriers to health care access among Canadians with chronic back pain (CBP) have been identified; however, no study has used lived experiences of people with CBP to drive the selection, analysis, and interpretation of variables most meaningful to patients.

**Objective:**

The aims of this study are to (1) engage with rural, remote, and urban Indigenous and non-Indigenous patients, health care providers, and health system decision makers to explore lived experiences among people with CBP in Saskatchewan, Canada; (2) cocreate meaningful indicators of CBP care access and effectiveness; and (3) identify program and policy recommendations to overcome access barriers to CBP care.

**Methods:**

In phase 1, one-on-one interviews with 30 people with current or past CBP and 10 health care providers residing or practicing in rural, remote, or urban Saskatchewan communities will be conducted. We will recruit Indigenous (n=10) and non-Indigenous (n=20) rural, remote, and urban people. In phase 2, findings from the interviews will inform development of a population-based telephone survey focused on access to health care barriers and facilitators among rural, remote, and urban people; this survey will be administered to 383 residents with CBP across Saskatchewan. In phase 3, phase 1 and 2 findings will be presented to provincial and national policy makers; health system decision makers; health care providers; rural, remote, and urban people with CBP and their communities; and other knowledge users at an interactive end-of-project knowledge translation event. A World Café method will facilitate interactive dialogue designed to catalyze future patient-oriented research and pathways to improve access to CBP care. Patient engagement will be conducted, wherein people with lived experience of CBP, including Indigenous and non-Indigenous people from rural, remote, and urban communities (ie, patient partners), are equal members of the research team. Patient partners are engaged throughout the research process, providing unique knowledge to ensure more comprehensive collection of data while shaping culturally appropriate messages and methods of sharing findings to knowledge users.

**Results:**

Participant recruitment began in January 2021. Phase 1 interviews occurred between January 2021 and September 2022. Phase 2 phone survey was administered in May 2022. Final results are anticipated in late 2022.

**Conclusions:**

This study will privilege patient experiences to better understand current health care use and potential access challenges and facilitators among rural, remote, and urban people with CBP in Saskatchewan. We aim to inform the development of comprehensive measures that will be sensitive to geographical location and relevant to culturally diverse people with CBP, ultimately leading to enhanced access to more patient-centered care for CBP.

**International Registered Report Identifier (IRRID):**

DERR1-10.2196/42484

## Introduction

Back pain is a common and costly health problem, as well as the leading cause of disability worldwide [1]. In Canada, 1 in 5 adults experience chronic back pain (CBP) [2], with associated health care costs estimated at US $6 billion to US $12 billion annually [3]. Canadians living in rural/remote areas are 30% more likely to experience CBP compared to urban dwellers, with Indigenous people reporting disproportionately higher rates [4]. CBP negatively impacts an individual’s quality of life and the health care system due to high rates of primary physician care visits [5], specialist consultations, diagnostic procedures [6], and opioid use [7]. Early access to physiotherapy care among people with back pain can result in up to 89% less likelihood of opioid prescription [8]. Therefore, improving access to nonpharmaceutical back pain treatment options such as physiotherapy is an especially important public health issue in Canada.

People living with CBP in Saskatchewan and Canada face diverse barriers to accessing physiotherapy services, which include the geographical location of the services, costs, and wait times [9-13]. In large geographic spaces like Saskatchewan, approximately 36% of the population live in rural settings; however, only 10% of the physiotherapists practice in these communities [12,14]. Approximately one-third of the Canadians do not have additional health insurance that would help to cover costs of care for treatments such as physiotherapy services [5], which are typically not covered through the provincial public health system. In conjunction with reduced access and limited resources to support publicly funded physiotherapy services, the lack of interprofessional team support in rural settings are key challenges identified by Canadian physicians [15]. In Saskatchewan, a prior evaluation of an urban-based spinal triage service (a collaborative practice model between orthopedic surgeons and physiotherapists developed to reduce wait times and address the problem of excessive referrals that were largely nonsurgical candidates) found that more than 70% of the patients referred to the service were from rural and remote communities [16]. Patients and providers of this service highlighted specific gaps in primary and rehabilitation care in rural and remote contexts, including reduced access to appropriate and timely care [11]. This is emerging as a critical challenge in Canada for individuals living with CBP.

In addition to non-Indigenous populations in rural and remote locations facing disparities in access, Indigenous populations experience unique inequities in their access to CBP care and are 30% more likely to experience CBP [4]. There is a paucity of data on the experiences of Indigenous peoples living with CBP in Canada. Within the scope of this paper, Indigenous refers to unique and distinctively different population groups, including First Nations (specifically Cree) and Métis peoples. Research examining CBP among Aboriginal people in Australia found that this condition can be profoundly disabling and that issues of sex and gender, cultural obligations, and emotional consequences are important considerations for health care [17]. Similarly, in Canada, a complex constellation of historical, psychosocial, cultural, and environmental factors influences general health and well-being among Indigenous people [4,18]. Racism and discrimination, rooted in Canada’s history of colonization and intergenerational trauma, play a significant role impacting disparities in access to health care and health outcomes, including CBP [19-22]. To redress these disparities and inequities, improve CBP outcomes, and inform culture- and strength-based strategies/interventions, we must work in partnership with and among Indigenous community members to obtain a greater understanding of the unique characteristics, strengths, needs, and challenges that are negotiated daily. Actively engaging Indigenous community members throughout the research process can ensure that Indigenous worldviews, language, culture, community practices, and protocols are followed and will inform how we can enhance and measure health care access and delivery in culture-based and meaningful ways.

Although the potential barriers to health care access among Canadians with CBP have been identified through population-based secondary data analyses [9] and through qualitative exploration of focused population groups (eg, farmers [23], patients receiving spinal triage service [24]), no known study in Canada has used the lived experiences of people with CBP to drive the selection, analysis, and interpretation of variables that are most meaningful to patients. Furthermore, no published studies have integrated Indigenous perspectives into identifying measures that will be relevant to informing future interventions, policies, and service provision to Indigenous peoples living with CBP. By integrating patients’ lived experiences of CBP and practitioners’ experiences providing care for people with CBP, this project will facilitate the identification and development of potential programs and policies to overcome access barriers. This work will focus on patient-identified measures that should be used to evaluate and inform the scale and spread of ongoing [25-27] and future community-based intervention studies to be more meaningful for patients and improve patient-reported outcomes.

The objective of this study is to engage with rural, remote, and urban Indigenous and non-Indigenous people with CBP, health care providers, and health system decision makers to (1) explore the lived experiences of access to health care among people with CBP in Saskatchewan as well as identify components unique to each group; (2) cocreate indicators of access to and effectiveness of back pain care that are most meaningful to people with CBP to inform evaluation of health care access interventions; and (3) identify programs or policy changes that could be implemented and evaluated in future, more comprehensive, and patient-oriented funding applications and projects.

## Methods

### Defining CBP

Low back disorders include a large group of clinical and etiological entities and there is no “gold standard” clinical classification or validated diagnostic criteria for many of these conditions [28]. Furthermore, the International Classification of Diseases-10 system does not have an adequate and distinct diagnostic code(s) for chronic pain or CBP [29]. Therefore, for this study, CBP includes self-reported pain and disability that lasted for a minimum of 3 months [30] that is related to low back injury (ie, sprain/strain) or low back pain with or without associated hip or leg symptoms due to pain referral.

### Defining Rural, Remote, and Urban

The metropolitan influenced zone (MIZ) classification developed by Statistics Canada was chosen for defining rural, remote, and urban residence because it is readily comparable to other Canadian research and considers not simply the geographic proximity but the degree of connectivity with urban areas [31,32]. Urban residence is classified as living in a town or city with ≥10,000 residents as determined on the basis of having a number other than 0 in the second position of the postal code. Rural status is defined as those communities outside of a census metropolitan area or a census agglomeration and include all MIZ categories: strong, moderate, or weak, as per Statistics Canada definitions [31]. Remote status comprises areas with no MIZ, which includes all census subdivisions that have a small, employed labor force (less than 40 people) as well as any census subdivision where no individuals commute to a census metropolitan area or a census agglomeration urban core [31]. Indigenous people living on reserve land will be further classified, since reserves are commonly situated in nonurban settings and may experience obstacles related to lack of access to health resources and community infrastructure that may be different from MIZ alone [18]. Due to the multifactorial nature of rural, remote, and urban status in terms of health care access, we will also ask participants how they perceive the meaning and definition of these geographic terms and other ways they define their home communities to further understand how these terms could be defined to be meaningful regarding access to health care.

### Patient-Oriented Approach

Patient-oriented research is the cornerstone to evidence-informed health care, referring to research processes informed by full and active involvement of patient partners in all aspects of the research [33]. The goal of engaging patients on the research team is to improve the translation of innovative approaches to ultimately ensure that the right patient receives the right clinical intervention at the right time [33]. The theoretical result of patient-oriented research is improved health outcomes. The Patient-Centered Outcomes Research Institute states that “The evidence base for stakeholder engagement in clinical research is growing; it shows that engagement is associated with increased recruitment and retention of study populations; more patient-centered and culturally appropriate methods; and greater relevance of research questions and outcome measures” [34].

In staying true to patient-oriented research processes, patient involvement in this project started with the identification of the research topic. Building on our existing work and community partnerships, the research topic of the outlined protocol was identified as a priority by patients through prior Saskatchewan-based research with non-Indigenous populations [10,11,23,27]. In addition, in a prior year-long community-based needs assessment of a remote Northern Saskatchewan Cree community, in partnership with Indigenous and non-Indigenous scholars, including people with CBP, Indigenous Elders, health care providers, and decision makers [25], participants described that CBP impacts a large proportion of the population and profoundly affects the physical, mental, and social quality of life of remote Indigenous patients.

Patient team members have already been recruited to this study. Team members were recruited through research team members’ networks and contacts. We currently have 2 non-Indigenous patient team members (1 rural and 1 urban) and 1 Cree team member (remote), all of whom have lived experiences with CBP. The role of our patient team members will involve close collaboration at every stage of the research, including identifying research priorities, objectives and questions, design, data collection, analysis, and dissemination. This will be accomplished through having dedicated time for open dialogue during team meetings that will privilege patient perspectives and voices. Further to this, research team leaders will follow up with patient team members individually after each meeting by using adaptive approaches of communication and engagement to best support their comfort levels and preferred methods of communication.

Layered strategies for respectful Indigenous engagement ensure that the research follows culturally appropriate and respectful processes and is directed by Indigenous perspectives. Researchers will follow the recommendations of the Tri-Council Policy Statement: Ethical Conduct for Research Involving Humans Chapter 9: Research Involving the First Nations, Inuit, and Métis Peoples of Canada [19] and principles of Ownership, Control, Access, and Possession [35]. The Indigenous patient team member has long-standing relationships with the research team and has been instrumental in guiding and grounding all aspects of this research in Cree knowledge, practice, and protocol. A Métis Elder also advised on community-wide education and participant recruitment.

Patient team members will be actively engaged to identify and refine the research outcomes specific to this project. Furthermore, patient participants recruited specifically for this project will be actively engaged to identify meaningful outcome measures and indicators pertaining to future CBP care intervention research.

Recruitment for people with CBP will occur primarily online through funding partner websites, university websites, and social media. We will use posters at health care provider clinics as well as team networks. Recruitment for health care providers will occur through research team and collaborator networks via posters, social media, as well as an email invitation for health care providers through targeted recruitment. For research team members who have pre-existing relationships with Indigenous community members, we will be looking to their guidance on other recruitment methods that will be unique to each community. For phase 1, potential participants will contact our research team to be screened for eligibility. They will be provided with the consent form and have the opportunity to review and ask questions prior to the interview. At the conclusion of the interview, informed consent will be confirmed. For phase 2, verbal consent will be obtained at the time of the phone survey.

### Interdisciplinary Team

Our diverse multidisciplinary research team includes Indigenous and non-Indigenous scholars, health care providers, and decision makers who have expertise and experience in health services research, collaborative models of care, musculoskeletal health, health economics, mixed methods, rural and remote health service delivery, integrated knowledge translation, Indigenous health, community-based participatory action health research approaches, health promotion intervention research, intergenerational and life course approaches to care and research, primary health care service delivery, and management. We also have team members that have already successfully led patient-oriented research projects and teams in rural, remote, and Indigenous communities. Inclusion of patient team members (2 non-Indigenous and 1 Indigenous from urban, rural, and remote communities) with unique knowledge, lived experience of CBP, language, and culture will be actively engaged in all stages of the research project. To complement and complete our team, we have connected with individuals and organizations as collaborators to help ensure the feasibility and relevance of our research and increase likelihood that findings will inform clinical practice, health care policies, and ultimately patient care/quality of care in a community-relevant and culturally respectful manner.

### Ethics Approval

The Behavioral Research Ethics Board at the University of Saskatchewan provided ethics approval (Beh 1973) for this project and the methods described.

### Procedures

**Figure 1:**
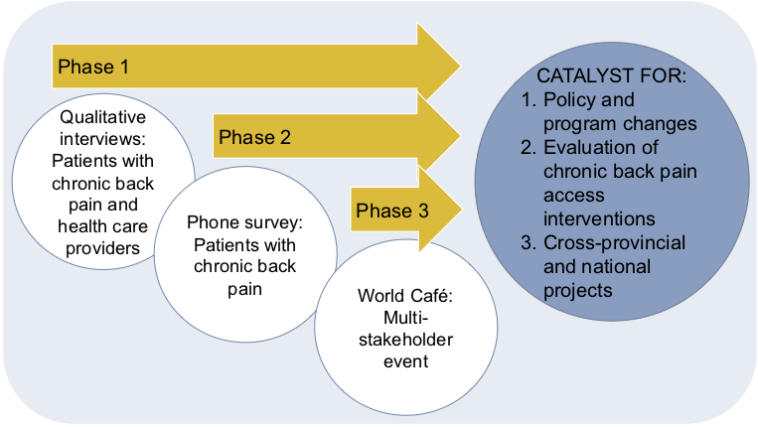
Project phases and outcomes.

This research will occur in 3 phases, with each phase informing the next (Figure 1).

#### Phase 1: Interviews and Web-Based Survey

In this phase, we will use a qualitative interpretive research paradigm, which aims to understand a phenomenon based on personal experiences, interpretations, and perceptions [36,37]. We will use purposive sampling to select a variety of people with CBP and health care providers. Participants will complete a closed web-based survey with a unique link provided for each participant. A completion check will be performed prior to the interview. We will conduct one-on-one interviews with approximately 30 people living with CBP (age>18 years with a minimum 3 months history of current or past low back pain) (Multimedia Appendix 1) and 10-12 health care providers who provide CBP care (including physicians, nurse practitioners, physiotherapists, occupational therapists, chiropractors, massage therapists, pharmacists, psychologists, and traditional healers) (Multimedia Appendix 2); all participants will reside or practice in rural, remote, or urban Saskatchewan communities and may or may not serve Indigenous communities. We will recruit both Indigenous (n=10) and non-Indigenous (n=20) people with CBP, with a goal of recruiting 10 people from each of the rural, remote, and urban communities. Data collection will be stopped at the point of saturation when no new information relevant to our research questions is generated [38]. Although the number of interviews will depend on the emerging analysis, it is anticipated that the proposed sample size for this phase is reasonable based on our prior qualitative research in this area [23,27,39]. We will perform interviews in-person (whenever feasible and preferred by participants and as allowed by public health guidelines surrounding the COVID-19 pandemic) or over telephone or videoconference. We will pay particular attention to the unique experiences shared by Indigenous participants living with CBP in rural, remote, and urban communities as guided by our Indigenous team members and collaborator partners. Interviews will be recorded, transcribed, and analyzed thematically using qualitative coding software (NVivo, QSR International) after obtaining consent from participants. Participants will have the opportunity to review transcripts prior to data analysis and synthesis. The information gained through these interviews will be examined together with the patient advisor team members at the analysis phase to guide the next stage of the project.

#### Phase 2: Population-Based Phone Survey

Findings from the interviews will be used to inform development of a population-based phone survey focused on exploring perceived health care access barriers and facilitators among 383 rural, remote, and urban residents with CBP. The same inclusion criteria as above will be used. Survey research specialists at the Canadian Hub for Applied and Social Research (CHASR) will co-design the survey with the research team (Multimedia Appendix 3). The survey will be deployed by CHASR. The estimated sample of 383 is based on the estimate that 20% of the adult population has CBP [2], the adult population of Saskatchewan (N=843,975) according to most recent census data [40], and a margin of error of +5.00%. Oversampling of rural and remote areas will be performed to reflect the higher prevalence of CBP [2,4]. The CHASR sampling methodology for telephone surveys includes obtaining samples from a third-party vendor (ASDE Survey Sampler), which obtains its panel through random digit dialing. To facilitate a more representative sample, both landline and mobile telephone numbers will be purchased. In accordance with the 2016 Communications Monitoring Report [41], 25.8% of the Saskatchewan households are mobile-only households and this will be reflected in the sample. Once the sample has been purchased, telephone numbers will be dialed randomly using the Voxco Computer Assisted Telephone Interviewing telephone survey software (Voxco, v.4.5). Telephone numbers are scheduled to be called up to 5 times without a response before the number is discarded. The survey items will be predominantly quantitative with some open-ended questions for qualitative analysis. Preliminary descriptive quantitative and qualitative analysis and reporting will be performed by analysts at the University of Saskatchewan’s CHASR with further analysis and interpretation undertaken by the research team (including patient team members). Stratification of survey findings by residence (ie, rural, remote, urban) and Indigenous self-identification will be undertaken to uncover patterns of perceived barriers and facilitators to access care identified by geographically and culturally diverse groups of people with CBP. In addition to examining patterns across geography and culture, survey analysis will include disaggregation by sex and gender-related variables (specific variables to be determined in conjunction with patient team members and informed by qualitative interviews).

#### Phase 3: Knowledge Translation and Community Engagement

An integrated knowledge translation approach will be utilized by engaging patients, health care providers, and decision maker team members throughout the research process and all phases of the project; however, phase 3 specifically focuses on end-of-project knowledge translation activities. Findings from phases 1 and 2 will be presented to provincial and national policy makers, health system decision makers, health care providers, urban rural and remote people with CBP, and other knowledge users at an interactive end-of-project knowledge translation event. This 1-day event will take place either in-person, virtually, or a combination of both, as public health guidelines surrounding the COVID-19 pandemic allow. Approximately 50 participants in addition to the research team will be invited to take part in this knowledge translation World Café, and will include rural, remote, urban, and Indigenous people with CBP, Indigenous Elders, health care managers, health care providers, and provincial and national decision makers. The morning of the event will include sharing the findings from the first 2 phases of the project. The afternoon will employ a World Café method [42] of facilitated dialogue that will serve to catalyze future patient-oriented research projects as well as actionable recommendations for policy and practice to improve pathways to accessing care for CBP. World Café is a collaborative and conversational process known to support knowledge exchange and creation, often through successive small group discussions [42]. Participants with different geographical, cultural, sex/gender, and role (ie, patient, health care provider, policy maker) backgrounds will be put into groups and will go through evolving rounds/café tables and discuss a range of topics. Each table will have a facilitator with experience in the given topic and will be responsible for taking notes as a form of data collection as well as to summarize and inform the next group discussion. As groups move through the rounds, discussions will elaborate on and enhance previous groups’ dialogues. This type of information exchange will allow participants to provide their unique perspectives and expertise on specific topic areas as well as to learn from the other participants. Through this method, relationships and connections will be made between participants with CBP, Indigenous and non-Indigenous community members, decision makers, and other stakeholders to create momentum and support for ongoing research, policy change recommendations, and promotion of the knowledge generated. Ideas and recommendations garnered from the World Café will be collected and synthesized by the project team members and culminate in a report to be shared with event participants and other relevant knowledge users who were not able to take part in the event. This event will allow for a participatory 2-way dialogue between the research team and knowledge users to help interpret the meaning and plan for the next steps of health service delivery planning, advocacy, and future research directions.

### Patients as Partners

Patient team members will contribute to conducting the research in the development of interview guides (phase 1), survey questions (phase 2), and recruitment strategies (phase 1 and 2); input in the analysis and interpretation of interview and survey findings (phase 1 and 2); and participation in the development of knowledge translation and exchange activities, including planning and participating in the end of project knowledge translation event (phase 3). Their involvement will also include supporting recruitment at community levels and determination of appropriate messages as well as identification of target audiences and methods of sharing the findings to knowledge users (people with CBP, decision makers, health care providers, the public at large).

### COVID-19–Related Methodological Considerations

Our team has had to make adaptations to our research plan and approach considering the current and evolving local COVID-19 public health measures and guidelines. Our team meetings to date, including communication with patient team members, have been held virtually. Further, we have adapted all data collections in phase 1 and 2 to be conducted virtually (ie, videoconference, online, or phone) at this time. It is unclear at this point if phase 3 will be required to be done entirely virtually, in-person, or some combination of both approaches. Further alterations to our research have included consideration of how the COVID-19 pandemic has impacted perceived access to CBP care through the addition of specific questions regarding this issue in phase 1 and 2 of the project.

## Results

This study was funded on February 2020. Research ethics board approval was received on September 4, 2020, following reopening of closures caused by the COVID-19 pandemic and research restart processes. Rolling participant recruitment started in January 2021. Interviews began in January 2021 and were completed in September 2022. Surveys and interviews of 33 patients and 16 health care providers were conducted. The population-based phone survey (phase 2) was administered between May 5 and 25, 2022 and preliminary descriptive quantitative and qualitative analyses and reporting data analyses were received from CHASR on July 21, 2022. Final analysis will be completed by November 2022. The anticipated barriers and facilitators for access to care for people with CBP are expected to be identified, with overlapping as well as distinct themes across rural, remote, urban and Indigenous populations, with some unique to each population as well.

## Discussion

### Anticipated Outcomes and Considerations

This research project is timely amid persisting health equity gaps in rural, remote, urban, and Indigenous populations, the current opioid crisis, and the potential impact of the COVID-19 pandemic on health and access to care [7,8,18,43,44]. We anticipate the outcomes of this project will have immediate and long-term impacts on patient-oriented research and patient-centered care. Engaging patients and other stakeholders as partners in research is recognized as a promising approach to generate evidence that is trusted, meaningful, and useful to clinicians, patients, and their families when making health care decisions [34]. The design of this project and engagement with patients and other stakeholders will build our capacity to engage in future meaningful patient-oriented research endeavors respectfully and effectively. Patient team members will benefit by engaging in a respectful dialogue about their CBP health needs and by actively guiding how they would like to see care delivered and evaluated. It will advance knowledge of the access barriers and facilitators to CBP care as identified by rural, remote, and urban residents. Findings will reveal factors that uniquely impact CBP care among Indigenous communities and peoples, further highlighting important culture-based considerations that will impact policy, resource, and implementation practices required to meet the health needs of diverse Indigenous populations living in Saskatchewan. This research will be shared with provincial and federal decision makers to help inform policies and strategies to enhance health service accessibility for CBP in rural, remote, urban, and Indigenous communities. The findings from this research will be shared through community and stakeholder presentations, academic conferences and networks, and peer-reviewed publications.

Patient and provider partnerships in this research will allow for the examination of priorities and concerns of those working within and relying on the health system services for CBP care. Working with Indigenous and non-Indigenous people with CBP from rural, remote, and urban locations will provide patient and community perspectives on CBP care gaps and needs. This is particularly important for Indigenous peoples who are required to navigate 2 worldviews in their access to health care (the Western worldview upon which most health systems in Canada are based and their unique Indigenous worldview). Our project integrates Cree knowledge and perspectives throughout the research process, thereby ensuring that the Cree culture, language, practices, and protocols essential to Cree wellness guide this work. Researchers and practitioners must work in partnership with Indigenous people and take responsibility to create space to uplift Indigenous worldviews above those of Western [45,46]. This, in turn, may be relevant to other First Nations, Métis, and Inuit populations and has the potential to optimize care for and with Indigenous people and foster a health system that works to dismantle racism and discrimination. It is anticipated that this study will lead to the creation of more effective, accessible, and appropriate CBP care that is responsive to the unique needs of individuals living in rural and remote areas and Indigenous peoples living in Saskatchewan. The involvement of key decision-maker stakeholders in the project (ie, provincial and federal managers/directors and policy makers) will help to ensure that the learning from this project can be translated into the development of policies and services to enhance equitable access to care for CBP across the province and beyond.

### Strengths and Limitations

This project is designed to engage with multiple groups and knowledge users in the circle of patient care, including patients, providers, and policy and decision makers, thereby having the potential to contribute to health care systems and practices. The examination of patient and provider perspectives from rural, remote, and urban areas and Indigenous communities will create greater contextualization and allow for patient, geographic, and Indigenous wellness-specific factors to be addressed when it comes to examining how current services and models are addressing the needs of people living in Saskatchewan with CBP. Engaging Cree patient perspectives will support the integration of Cree epistemology and ensure that the principles of self-determination and self-governance are recognized and upheld. This research approach aims to address the issue of barriers and facilitators more holistically to access CBP care and highlight future areas to address and inform the health care system, clinical care guidelines, and other resources that are patient-oriented and community-directed.

The limitations of this research include potentially small subsample sizes that may not allow for specific comparisons between groups. In addition, the research findings may not be broadly generalizable, given that it has a Saskatchewan-specific focus.

### Future Direction

The information gained in this project will ultimately provide patient-driven perspectives and outcomes that will serve as a catalyst for future research in a health care environment where new and innovative approaches are needed to address challenges to accessing more effective and appropriate CBP care.

## Appendix

Multimedia Appendix 1 Interview guide for patients with chronic back pain.

Multimedia Appendix 2 Interview guide for health care providers.

Multimedia Appendix 3 Telephone survey template.

Multimedia Appendix 4 Peer review report by Canadian Institutes of Health Research / Instituts de recherche en santé du Canada (CIHR/IRSC) - Catalyst Grant: Patient-Oriented Research/Subvention catalyseur : Recherche axée sur le patient (Canada).

## Abbreviations

CBP: chronic back pain
CHASR: Canadian Hub for Applied and Social Research
MIZ: metropolitan influenced zone

## Data Availability

The data sets generated and analyzed during this study are available from the corresponding author on reasonable request.
