# Supplementary material for: Experiences of Health Care Access Challenges for Back Pain Care Across the Rural-Urban Continuum in Canada: Protocol for Cross-sectional Research
Source: JMIR Res Protoc. 2022 Dec 19;11(12):e42484. doi: 10.2196/42484 (PMC9808614; doi:10.2196/42484)
Supplement: Multimedia Appendix 1 [file resprot_v11i12e42484_app1.docx]

**Interview Guide for People with Chronic Back Pain**

***Please note that these questions will be reviewed and further informed by our patient team members, as they may have other suggested questions/modifications to questions***

**Preamble:**

Hello, my name is X, and I am part of the research team looking how easy or difficult it is for people with back pain to get the help and care they need. Thank you for agreeing to talk to me about your experiences with having low back problems.

As you know from the consent form we just reviewed together, the information you share today will help us to better understand: your personal experiences with back pain (both positive and negative), and ways we might be able to measure and understand future changes to accessing healthcare in your community.

The information you share will help us to better understand what care you need for your back pain at the time that you need it, which may also be helpful to other people in a similar situation.

Your information will be combined with information shared by others from across Saskatchewan who have also experienced low back pain, as well as health care providers for those with low back pain (like doctors, nurses, or physiotherapists for example). We plan to share our findings with policy and decision makers, with the hope of improving access to healthcare services for you and other residents of Saskatchewan.

We want to acknowledge the environment that we are in today with Covid-19. Your experience today may be very different from your initial experiences in accessing healthcare for your back pain. During our interview, try to think of your long-term experiences and the big picture, and not just what is going on today. We know that the last few months will have influenced your experience, and we would like to hear about your full experience, so hopefully our questions will allow you to share your full experience.

This interview/ discussion should take approximately 30 minutes to 1 hour. As we reviewed in the consent form, I will be recording our conversation today and the recording will then be typed up into a written script for analysis after the research project is completed. Are you ok with proceeding with the interview at this time?

Do you have any questions before we get started?

*If no...*

Let's get started...I am turning on the recorder now.

1. Tell me a bit about your back pain. *(pause after this statement and wait for a response; and if needed, use the following bulleted list as probes to draw out information from the participant)*

2. Can you tell me about when your back pain started?

a) Has it been constant since it started, or has it come and gone?

2. How has having back pain affected your life?

Prompts:

a) Physical abilities?

b) Social participation? i.e. activities with family, friends, community, work

c) Emotional consequences?

d) Your ability to practice your cultural and/or spiritual activities?

e) Other?

f) Has this changed since COVID-19?

2. What types of supports/ services have you tried for your back pain? What has been

helpful?

Prompts:

a) Health care services? (including physical therapy services...? Medications?)

b) Community supports?

c) Others? (e.g. companion animals)

d) Local/traditional cultural practices?

3. Can you tell me about any challenges you have had in trying to access health care for your back problems?

Prompts:

a) Travel?

b) Wait times?

c) Financial/ costs?

i. Need for accommodation while away? Food and/or per diem? Food reimbursement adequate for your needs?

ii. Ability to pay for a companion to join you for your appointments?

d) Cultural?

e) Comfort interacting with health care professionals?

f) Other?

(Additional prompt considering services responses above):

*Which services were impacted by these challenges?*

*Have this changed since COVID-19?*

4. What types of other services and/ or supports do you think would help to support you in better treating and managing your low back pain and overall abilities to do the things you want to do?

5. Can you tell me about things that have helped you get the care you need for your back problems?

Prompts:

a) For example, something that helped remove financial barriers, or geographic/location barriers, wait times or cultural mismatch?

b) extra health insurance?

c) publicly funded service?

d) care in the community?

e) quick access to health care providers?

f) others?

6. Can you tell me about a time when you got care for your back problems and it turned out well?

Prompts:

a) What was good about it?

b) What about that experience made it helpful?

c) Anything that could have improved it even more?

d) Are there any other experiences you can share, where you had a positive experience?

7. Can you tell me about a time when you got care for your back problems, and it did NOT turn out well?

Prompts:

a) What was negative about it?

b) What about that experience made it negative?

c) Anything that could have improved your experience?

d) Are there any other experiences you can share, where you had a negative experience?

8. What would help make it easier for you to access healthcare?

9. What are things that are meaningful or important to you when it comes to improving access to care for your back problems?

Prompts (Use terms in brackets):

a) affordability (costs, travel time, how you are able to pay for services)

b) availability and accessibility (location of services, hours of opening, appointment mechanisms, short wait times)

c) Appropriateness (Quality of care, trusting relationship, effectiveness of care)

d) Acceptability (professional values/beliefs are in line with your own, cultural awareness/understanding/connection, gender)

e) Approachability (transparency, services that make themselves known and use language that is understandable)

10. If changes in health care services occurred in your community, do you have any suggestions on how we could measure if these changes were successful in improving care?

*Pause and use the following prompts if needed:*

1. The experiences/ stories of people with back pain?

2. Less Pain? Measuring pain levels?

3. Better quality of life? (Clarifying what types of activities you are now able to participate in, that you couldn’t do before receiving appropriate health care...?)

4. Better movement/ mobility?

5. More able to participate in social/ community activities?

6. Less use of prescription medicines?

7. Less travel from the community?

8. Others?

11. Over the past several months, healthcare practices have been forced to change due to COVID-19. How have these changes impacted your ability to access the care that you’ve needed for your back?

Prompts:

1. Have you been able to continue receiving care?
2. Has the way in which you’ve received care changed? How?
3. What has been difficult about these changes?
4. What has been easier for you in accessing health care providers? (ie –appointments being held over the phone or videoconference)

12. Is there anything else you would like to share with us about either your experiences with back problems, health care access or use of health care services?

a) (Once they stop talking) anything else we should know?

Thanks so much for your time and your thoughts. Before we finish today, I would like to go back to the consent form briefly. Now that we have been through the interview and you know what you have shared with me, I just want to go back through the sections we checked off and see if you still consent in the same way as before we started. It’s perfectly OK to change your mind on any of this. [At this point confirm all the check box decisions with the participant]
