# Supplementary material for: Experiences of Health Care Access Challenges for Back Pain Care Across the Rural-Urban Continuum in Canada: Protocol for Cross-sectional Research
Source: JMIR Res Protoc. 2022 Dec 19;11(12):e42484. doi: 10.2196/42484 (PMC9808614; doi:10.2196/42484)
Supplement: Multimedia Appendix 2 [file resprot_v11i12e42484_app2.docx]

**Interview Guide for Health care providers**

Preamble:

Hello, my name is X, and I am part of the research team looking at health care access challenges for back pain management across rural, remote, and urban regions in Saskatchewan.

Thank you for agreeing to talk to me and share your guidance and experiences. The information you provide will help us to better understand: your observations of experiences of your patients or community members in coping with chronic back pain (both positive and negative) and what type of measures would be relevant to you and/ or the community to help understand appropriate access to health care for chronic back pain.

This information you provide will help us to better understand what the local needs are for chronic back pain care. Your information will be combined with information shared with patient experiences in Saskatchewan who have experienced low back pain, as well as other health care providers, to share with policy and decision makers, with the hope of improving access to health care services across Saskatchewan.

We want to acknowledge the environment that we are in today with COVID-19. Your experience today may be very different from your pre-COVID-19 experiences in providing healthcare for chronic back pain. During our interview, try to think of your long-term experiences and the big picture, and not just what is going on today. We know that the last few months will have influenced your practice, and we would like to hear about your full experience, so hopefully our questions will allow you to share your full experience.

This interview/ discussion should take approximately 30 minutes to 1 hour. As we reviewed in the consent form, I will be recording our conversation today and the recording will then be typed up into a written script for analysis after the research project is

completed.

Are you ok with proceeding with the interview at this time?

Do you have any questions before we get started?

*If no...*

Let's get started...I am turning on the recorder now.

1. Tell me a bit about what you have observed regarding your patients who experience persistent low back pain? *(pause after each statement and wait for a response; and if needed, use the following bulleted list as probes to draw out information from the participant)*

2. How has having back pain affected the lives of your patients/ clients

and people in your community?

Prompts:

a) Physical abilities?

b) Social participation? i.e. activities with family, friends,

community, work

c) Emotional consequences?

d) The ability to practice culture and/or spiritual activities?

e) Other?

f) Do you think this has changed since Covid-19?

3. What types of supports/ services do you think would be helpful to improve management of your patients/ clients with chronic back pain and people with chronic back pain in your community?

Prompts:

a) Augmented Health care services (e.g. team care, access to other types of services in the community...?

b) Medications?

c) Pain management counselling etc.

d) Community supports? (e.g. local programs or facilities for physical activity etc.)

e) Others? (e.g. companion animals)

3. Are there additional/different supports/services that you think would be helpful for management of back pain for the Indigenous patients you see?

Prompts:

a) Local/traditional practices, ceremonies?

b) access to Elders/ knowledge keepers

c) care in their language/ access to interpreters

4. Can you tell me about any challenges your patients with CBP and people in the communities you serve have experienced in trying to access health care for back problems?

Prompts (Use terms in brackets):

a) affordability (costs, travel time, how they are able to pay for services)

b) availability and accessibility (location of services, hours of opening, appointment mechanisms, short wait times)

c) Appropriateness (Quality of care, trusting relationship, effectiveness of care)

d) Acceptability (values/beliefs are in line with your own, cultural differences, gender)

e) Approachability (transparency, services that make themselves known and use language that is understandable)

f) How has this changed since Covid-19?

5. What are barriers or challenges for you as a healthcare provider in providing optimal care for your patients with low back pain?

Prompts:

a) Available services in the area

b) Length of appointment time

c) Continuity in care?

d) Knowledge of cultural practices

e) Patient’s willingness/ability to consider/attend augmented health care services

6. What types of services and/ or supports do you think would help to overcome the challenges you indicated for your patients or for yourself as the healthcare provider?

7. What are existing community supports and strengths in the community/ communities that you serve that help to support people with back pain?

8. How would we know if changes in current services/accessibility were relevant and useful for people with back pain and the community/ communities as a whole? What types of measurements would help capture this?

Prompts:

a) Participant experiences/ stories?

b) Less pain?

c) Better quality of life?

d) Better movement/ mobility?

e) More able to participate in social/ community activities?

f) Less use of prescription medicines?

g) Less travel from the community?

h) Others?

9. Is there anything else you would like to share with us about either your observations of people with back problems, or health care access/ use?

10. Over the past several months, healthcare practices have been forced to change due to COVID-19. How have these changes impacted your ability to provide care for your patients with low back pain?

Prompts:

a) Have you continued to provide care for your patients with low back pain?

b) Has the way in which you’ve provided care changed? How?

c) What has been difficult about these changes?

d) What has been easier for you in providing health care during this time? (ie –appointments being held over the phone or videoconference)

e) What is your perception on how this has affected your patients?

Thanks so much for your time and your thoughts. Before we finish today, I would like to go back to the consent form briefly. Now that we have been through the interview and you know what you have shared with me, I just want to go back though the sections we checked off and see if you still consent in the same way as before we started. It’s perfectly OK to change your mind on any of this. [At this point confirm all the check box decisions with the participant]
