# Supplementary material for: Experiences of Health Care Access Challenges for Back Pain Care Across the Rural-Urban Continuum in Canada: Protocol for Cross-sectional Research
Source: JMIR Res Protoc. 2022 Dec 19;11(12):e42484. doi: 10.2196/42484 (PMC9808614; doi:10.2196/42484)
Supplement: Multimedia Appendix 3 [file resprot_v11i12e42484_app3.docx]

**CHRONIC BACK PAIN TELEPHONE SURVEY**

**(March 2022)**

**INTRODUCTION**

INT02.

Hello, my name is  ***(FIRST NAME ONLY)*** and I am a student calling from the University of Saskatchewan. We are conducting a short ten-minute telephone survey to explore the perceived health care access barriers and facilitators among people with chronic back pain in Saskatchewan. The purpose is to learn about some of the strengths and weakness to accessing health care in Saskatchewan.

INTRO2.

Is there anyone in your household who is 18 years of age or older and who is experiencing lower back pain that has lasted at least 3 months?

1. Yes, speaking ***CONTINUE***

2. Yes, I’ll get him/her ***REPEAT INTRODUCTION AND CONTINUE***

3. Not available ***ARRANGE CALLBACK 🡪 HIT “ESC” ON YOUR KEYBOARD –***

***REQUEST RESPONDENT FIRST NAME AND ARRANGE CALLBACK***

4. Refused to Transfer

INT03.

I would like to invite you to participate in this short survey. The name of the project is: *Living with Chronic Back Pain: A patient-led investigation of health care access challenges for back pain across the rural-urban continuum in Saskatchewan*. Participation is voluntary, and you can stop the survey at any time at which point your data will be deleted and destroyed. You can skip any questions you don’t want to answer. This call will be recorded for quality control purposes. Your responses will be combined anonymously with others once the call ends.   None of the answers that you provide will be linked back to you personally. There are no known risks or personal benefits to participating in this survey. Data will be kept and stored for a minimum of 5 years after results have been published on password-protected university managed systems (i.e OneDrive and DataStore) accessible by the research team. This research project has been approved on ethical grounds by the University of Saskatchewan Behavioural Research Ethics Board. Any questions regarding your rights as a participant may be addressed to that committee through the Research Ethics Office at [ethics.office@usask.ca](mailto:ethics.office@usask.ca) or 1-888-966-2975 If you have any questions or concerns about the survey itself, you may contact the lead researcher Dr. Brenna Bath at [brenna.bath@usask.ca](mailto:brenna.bath@usask.ca) or 306-966-6573.

Are you willing to participate?

1. Yes ***CONTINUE***
2. No ***THANK AND END INTERVIEW***
3. Later/Not right now ***ARRANGE CALLBACK*** 🡪 ***HIT “ESC” ON YOUR KEYBOARD –***

***REQUEST RESPONDENT FIRST NAME AND ARRANGE CALLBACK***

SCREEN1.

To confirm, you experience lower back pain? That is, pain localized between the bottom of the rib cage and the bottom of the buttocks, that may or may not include leg pain?

1. Yes
2. No ***THANK AND END INTERVIEW***
3. (Refused) ***THANK AND END INTERVIEW***

SCREEN2.

How long have you had problems with your low back? Was it…?

***(READ LIST)***

1. Less than 3 months ***THANK AND END INTERVIEW***
2. 3 to less than 6 months
3. 6 months to less than a year
4. 1 year to less than 5 years
5. 5 years to less than 10 years
6. 10 years or more
7. (Refused) ***THANK AND END INTERVIEW***

POSTAL.

Can I please have your postal code?

***IF RESPONDENT IS RELUCTANT, YOU CAN ASSURE THEM THAT THEIR POSTAL CODE WILL BE USED FOR STATISTICAL PURPOSES ONLY (TO UNDERSTAND DIFFERENCES BY REGION/GEOGRAPHY) AND WILL NOT BE USED TO IDENTIFY THEM IN ANY WAY.***

***ENSURE RESPONDENT PROVIDES POSTAL CODE IN PROPER FORMAT (EXAMPLE : S7N 1G4).***

***ENTER RESPONDENTS POSTAL CODE CHARACTERS:***

1. (RECORD POSTAL CODE)

INSTR1.
We’re now going to read you a list of health care providers. Please let us know if you have ever accessed care from them for your back pain. If you haven’t, we’ll ask you to indicate why. Possible reasons may include cost, wait time, travel time or location, you simply weren’t interested in accessing service from that provider, or you can provide us with another reason.

***(Interviewer note: is the respondent indicates they have not seen the health care provider but does not want to provide a reason or does not know the reason, please select “No, other” and type “refused” or “don’t know” respectively. Note the questions Q1A to Q1I will be asked in a random order.)***

Q1A.
Your family physician

1. Yes
2. No, cost
3. No, wait time
4. No, travel time/location
5. No, not interested
6. No, other *(SPECIFY)*
7. (Don’t Know)
8. (Refused)

Q1B.
A physiotherapist

1. Yes
2. No, cost
3. No, wait time
4. No, travel time/location
5. No, not interested
6. No, other *(SPECIFY)*
7. (Don’t Know)
8. (Refused)

Q1C.
A chiropractor

1. Yes
2. No, cost
3. No, wait time
4. No, travel time/location
5. No, not interested
6. No, other *(SPECIFY)*
7. (Don’t Know)
8. (Refused)

Q1D.
A massage therapist

1. Yes
2. No, cost
3. No, wait time
4. No, travel time/location
5. No, not interested
6. No, other *(SPECIFY)*
7. (Don’t Know)
8. (Refused)

Q1E.
An acupuncture provider

1. Yes
2. No, cost
3. No, wait time
4. No, travel time/location
5. No, not interested
6. No, other *(SPECIFY)*
7. (Don’t Know)
8. (Refused)

Q1F.
An Indigenous or traditional healer

1. Yes
2. No, cost
3. No, wait time
4. No, travel time/location
5. No, not interested
6. No, other *(SPECIFY)*
7. (Don’t Know)
8. (Refused)

Q1G.
A surgeon/specialist

1. Yes
2. No, cost
3. No, wait time
4. No, travel time/location
5. No, not interested
6. No, other *(SPECIFY)*
7. (Don’t Know)
8. (Refused)

Q1H.
An exercise therapist

1. Yes
2. No, cost
3. No, wait time
4. No, travel time/location
5. No, not interested
6. No, other *(SPECIFY)*
7. (Don’t Know)
8. (Refused)

Q1I.
Community supports, services, or facilities

1. Yes
2. No, cost
3. No, wait time
4. No, travel time/location
5. No, not interested
6. No, other *(SPECIFY)*
7. (Don’t Know)
8. (Refused)

Q1J.
Somewhere else?

***(Interview note: If respondent discusses barriers in their response, please ensure that information is recorded)***

1. (ENTER RESPONSE VERBATIM)
2. (Don’t Know)
3. (Refused)

Q2A.
Have you taken medication to treat your back pain?

1. Yes
2. No
3. (Don’t Know)
4. (Refused)

Q2B.
Have you used traditional healing methods to treat your back pain?

1. Yes, what type? (SPECIFY)
2. No
3. (Don’t Know)
4. (Refused)

INSTR3.
I’m now going to read you a list of possible factors that may or may not make it difficult for you to get the help you need for your back pain. Please let me know if the factor is not at all a barrier for you, a minor barrier, a moderate barrier, or a serious barrier to getting the help you need.

Travel to access health services

1. Not at all a barrier
2. A minor barrier
3. A moderate barrier
4. A serious barrier
5. (Don’t Know)
6. (Refused)

Q3B.
Wait times

1. Not at all a barrier
2. A minor barrier
3. A moderate barrier
4. A serious barrier
5. (Don’t Know)
6. (Refused)

Q3C.
Costs

1. Not at all a barrier
2. A minor barrier
3. A moderate barrier
4. A serious barrier
5. (Don’t Know)
6. (Refused)

Q3D.
Cultural sensitivity

1. Not at all a barrier
2. A minor barrier
3. A moderate barrier
4. A serious barrier
5. (Not Applicable)
6. (Don’t Know)
7. (Refused)

Q3E.
Comfort interacting with health care professionals

1. Not at all a barrier
2. A minor barrier
3. A moderate barrier
4. A serious barrier
5. (Don’t Know)
6. (Refused)

Q3F.
Personal responsibilities for providing care to others

1. Not at all a barrier
2. A minor barrier
3. A moderate barrier
4. A serious barrier
5. (Don’t Know)
6. (Refused)

Q3G.

Not knowing where to go or what will help (i.e. feeling lost in the health care system)

1. Not at all a barrier
2. A minor barrier
3. A moderate barrier
4. A serious barrier
5. (Don’t Know)
6. (Refused)

Q4.
Are there any other barriers you encounter that make it difficult for you to access care for your back pain?

1. YES (RECORD RESPONSE VERBATIM)
2. No
3. (Refused)

INSTR5.
I’m now going to read you a list of possible factors that may or may not make it easier for you to get the care you need for your back pain. Please let me know if the factor is not at all a help for you, a minor help, a moderate help, or a major help to getting the care you need for your back pain.

Q5A.Publicly funded health care

1. Not at all a help
2. A minor help
3. A moderate help
4. A major help
5. (Don’t Know)
6. (Refused)

Q5B. Health care nearby in your community.

1. Not at all a help
2. A minor help
3. A moderate help
4. A major help
5. (Don’t Know)
6. (Refused)

Q5C.Quick access to health care providers (i.e. no or short wait time)

1. Not at all a help
2. A minor help
3. A moderate help
4. A major help
5. (Don’t Know)
6. (Refused)

Q5D.Health care provider who helps you get care (i.e. refers you on for care or follow up)

1. Not at all a help
2. A minor help
3. A moderate help
4. A major help
5. (Don’t Know)
6. (Refused)

Q5E.Having additional health care insurance.

1. Not at all a help
2. A minor help
3. A moderate help
4. A major help
5. (Don’t Know)
6. (Refused)

Q5F.Telehealth/ virtual care

1. Not at all a help
2. A minor help
3. A moderate help
4. A major help
5. (Don’t Know)
6. (Refused)

Are there any other factors that have made it easier for you to access care for your back pain?

1. YES (RECORD RESPONSE VERBATIM)
2. No
3. (Refused)

Q6.
Over the past year or two, healthcare practices have changed due to COVID-19. Would you say these changes have impacted you positively, negatively, or had no impact on your ability to access the care you need for your back pain?

1. Positively
2. No impact
3. Negatively
4. (Not Applicable – did not try to access services during this time)
5. (Don’t Know)
6. (Refused)

Q6A- (If positive or negative) can you tell me WHY or HOW COVID-19 has impacted your ability to access the care you need? (RECORD RESPONSE VERBATIM)

D1.
Now to ensure that we are talking to a representative cross section of Saskatchewan residents, we need to get a little information about your background.

In what year were you born?

1. (ENTER YEAR OF BIRTH)

2. (Refused)

D2.
With what gender do you identify?

1. Man
2. Woman
3. I prefer to self-describe as: _______
4. Prefer not to answer

D3.
What is your height in feet and inches?

1. (RECORD RESPONSE)
2. (Refused)

D4.
What is your weight in pounds?

1. (RECORD RESPONSE)
2. (Refused)

D5.
Do you self-identify as Indigenous (First Nation, Métis, or Inuit)?

1. Yes
2. No ***SKIP TO Q6***
3. (Refused) ***SKIP TO Q6***

D5A.
Do you live on reserve land?

1. Yes
2. No
3. (Refused)

D5B.
Are you a traditional Knowledge Keeper or Elder?

1. Yes
2. No
3. (Refused)

D6.
What is the highest level of education that you have completed?

1. Did not complete Grade 12
2. High School
3. Trade/Technical School/College
4. Some University
5. Bachelor’s degree
6. Graduate Degree
7. (Refused)

D7.

Considering your main form of work, are you currently working full time, part time, a student, retired, caring for a child or family member, unemployed, disabled, engaged in cultural or traditional work, or something else?

1. Work full-time
2. Work part-time
3. Caring for a child or family member **SKIP TO D8**
4. Unemployed **SKIP TO D8**
5. Disabled, unable to work **SKIP TO D8**
6. Student **SKIP TO D8**
7. Retired **SKIP TO D8**
8. Cultural/traditional work (EXPLAIN) **SKIP TO D8**
9. Other (EXPLAIN) **SKIP TO D8**
10. (Refused) **SKIP TO D8**

D7A.
What is your occupation?

1. (RECORD OCCUPATION) **SKIP TO D9**
2. (Refused) **SKIP TO D9**

D8.
Are you not working because of your low back problem?

1. Yes
2. No
3. (Refused)

D9.
Do you live with someone who can support you in dealing with your back pain?

1. Yes (record relationship)
2. No
3. (Refused)

D10.
Do you have extra insurance coverage for health care in addition to Saskatchewan Health coverage (i.e. doctors, prescriptions and in hospital care)? If yes, what type is it?

***(READ LIST IF NECESSARY)***

1. Group Health insurance (ie – coverage through work health plan, Blue Cross, other)
2. SGI funding
3. Workers Compensation Board funding
4. Short or long term disability funding
5. Other funding
6. No additional coverage
7. (Don’t Know)
8. (Refused)

D11.
Could you please tell me your total annual household income from all sources in 2021. I can read you a list and just let me know where you fall.

***IF ASKED, ALL SOURCES INCLUDE EMPLOYMENT INCOME (WAGES OR SALARY), SAVINGS, PENSIONS, RENT, ETC.***

1. Less than $15,000
2. $15,000 to less than $30,000
3. $30,000 to less than $60,000
4. $60,000 to less than $100,000
5. $100,000 or more
6. (Don't Know)
7. (Refused)

E1. The researchers have another part of the study where they are doing one-on-one interviews for around 30 minutes about experiences with back pain and accessing care. Honoraria will be provided. The information you provided to me today will not be linked to your interview responses. The researchers are hoping to recruit more Indigenous participants and/ or people from remote communities. If you agree, I will collect your name and phone number and pass it on to the research team who may contact you at a later date. Would you be interested in taking part in a follow-up one-on-one interview?

1. Yes
2. No

(**If Yes, Record NAME, PHONE NUMBER**)

END.

Those are all the questions that I have! On behalf of the University of Saskatchewan, thank you for your time. Your responses are greatly appreciated! Have a great day/evening!
