# Supplementary material for: Experiences of Health Care Access Challenges for Back Pain Care Across the Rural-Urban Continuum in Canada: Protocol for Cross-sectional Research
Source: JMIR Res Protoc. 2022 Dec 19;11(12):e42484. doi: 10.2196/42484 (PMC9808614; doi:10.2196/42484)
Supplement: Multimedia Appendix 4 [file resprot_v11i12e42484_app4.pdf]

Canadian Institutes of Health Research
160 Elgin Street, 9th Floor
Address Locator 4809A
Ottawa, Ontario K1A 0W9

Instituts de recherche en santé du Canada
160, rue Elgin, 9e étage
Indice de l'adresse 4809A
Ottawa (Ontario) K1A 0W9

Institute of Aging
Institute of Cancer Research
Institute of Circulatory and Respiratory Health
Institute of Gender and Health
Institute of Genetics
Institute of Health Services and Policy Research
Institute of Human Development and Child and Youth Health
Institute of Indigenous Peoples' Health
Institute of Infection and Immunity
Institute of Musculoskeletal Health and Arthritis
Institute of Neurosciences, Mental Health and Addiction
Institute of Nutrition, Metabolism and Diabetes
Institute of Population and Public Health

Institut du vieillissement
Institut du cancer
Institut de la santé circulatoire et respiratoire
Institut de la santé des femmes et des hommes
Institut de génétique
Institut des services et des politiques de la santé
Institut du développement et de la santé des enfants et des adolescents
Institut de la santé des Autochtones
Institut des maladies infectieuses et immunitaires
Institut de l'appareil locomoteur et de l'arthrite
Institut des neurosciences, de la santé mentale et des toxicomanies
Institut de la nutrition, du métabolisme et du diabète
Institut de la santé publique et des populations

January 30, 2020

Dr. Brenna Lea Bath
School of Rehabilitation Science
University of Saskatchewan
104 Clinic Place
Saskatoon, Saskatchewan S7N 2Z4

Dear Dr. Bath:

On behalf of the Canadian Institutes of Health Research (CIHR), I wish to congratulate you on your success in the recent CIHR funding competition!

As health researchers, we are united by a common goal: to improve the health and well-being of Canadians and people throughout the world. Through CIHR, the Government of Canada provides vital support to Canadian researchers, spanning the tightly linked pillars of health research, with the ultimate goal of improving health for all Canadians. As a recipient of this funding, you are now part of this endeavour.

As you are aware, the evaluation of your grant application was made possible thanks to peer reviewers who generously volunteer their time to support the Canadian health research enterprise. As a CIHR-funded researcher, your knowledge and expertise are invaluable to this process. If you are not already a peer reviewer, I would like to take this opportunity to invite you to consider becoming a member of the College of Reviewers. Please visit www.cihr-irsc.gc.ca/e/49923.html to learn about the eligibility criteria and how to apply for membership.

As health researchers, we are living at a time when science is under careful scrutiny, and it has become more important than ever to ensure that our work is clearly understood. As such, I would ask that you recognize your CIHR funding when delivering presentations and communicating about your research, and that you continue to advocate for the critical importance of research in advancing the health of Canadians.

Once again, please accept my congratulations on this impressive achievement and my best wishes for success in all your endeavours. I look forward to following the progress of your research.

Sincerely,

Michael J. Strong, MD, FRCPC, FAAN, FCAHS
President

491738-201909PAO-PAO-425009-162598-CONGR

CIHR IRSC
Discoveries for life / Découvertes pour la vie

Canada

**Review Type/Type d'évaluation:**	Committee Member 1/Membre de comité 1
**Name of Applicant/Nom du chercheur:**	Bath, Brenna Lea
**Application No./Numéro de demande:**	425009
**Agency/Agence:**	CIHR/IRSC
**Competition/Concours:**	2019-09-10 Catalyst Grant: Patient-Oriented Research/Subvention catalyseur : Recherche axée sur le patient
**Committee/Comité:**	Catalyst Grant : Patient-Oriented Research/Subvention catalyseur : Recherche axée sur le patient
**Title/Titre:**	Patient experiences of health care access challenges for back pain care across the rural-urban continuum

Assessment/Évaluation:

This application meets the criteria of the competition. The research team has multiple and diverse patient perspectives fully integrated multiple and diverse patient perspectives, including under represented populations. They have collaborations and research partners in two jurisdictions, SK and AB. The area of chronic lower back pain is a huge health concern with significant impact on the health care system.

Review Type/Type d'évaluation: Committee Member 2/Membre de comité 2
Name of Applicant/Nom du chercheur: Bath, Brenna Lea
Application No./Numéro de demande: 425009
Agency/Agence: CIHR/IRSC
Competition/Concours: 2019-09-10 Catalyst Grant: Patient-Oriented Research/Subvention catalyseur : Recherche axée sur le patient
Committee/Comité: Catalyst Grant : Patient-Oriented Research/Subvention catalyseur : Recherche axée sur le patient
Title/Titre: Patient experiences of health care access challenges for back pain care across the rural-urban continuum

---

Assessment/Évaluation:

Research Approach

This proposal responds exceptionally well to the objective of the funding opportunity which is to engage patients, family members and others in a really meaningful way to build a new research approach. The proposed work is to meaningfully engage community-dwelling older adults and health and social service providers in conceptualizing the research program. The commitment of co-design of the research questions, methods and outputs in this proposal are very strong and this proposal demonstrates a deep understanding of the objectives of this research funding opportunity. This proposal demonstrates the team's sophisticated understanding of patient and citizen engagement and co-design. There is real and meaningful engagement in the proposed work.

The proposed work is very clear and it is very important. In fact it is quite relevant to SPOR in the sense of potentially improving health systems and health outcomes. There is meaningful engagement of people with lived experience in the proposed methods. There has been extensive work undertaken to date to engage those with lived experience into the development of the research issue. The team has previous experience in both the subject matter and co-design with people with lived experience.

People with lived experience have been and will be meaningfully engaged in identifying the topic of the work, the methods and the products of the research. The team has put together a thoughtful and broad recruitment strategy largely based on previous engagement with the study population. From identifying the research priorities and outcomes to the methodology. People with lived experience will play a central role in co-designing the research intervention. More specifics as to how the research team will undertake specific recruitment strategies to mitigate and address inequities would be hlpful. There is stated intent here but not a lot of detail as to how that will actually occur. But one assumes that the strong understanding of co-design and the meaningful engagement of those with lived experience may enable this outcome to be realized. The team could have explained this particular strategy to address inequities in more detail in the proposal. The team is strong academically speaking. It is also strong from a multi-disciplinary perspective and in working with those with lived experience.

There is the opportunity for this work to very meaningfully impact health systems practice and policy. The work could also help address health inequities and ensure more inclusive community design that enables more active, healthy living for older adults.

Sex and gender has been integrated into the proposal.

Feasibility

This work is very feasible and builds on previous work. It seeks to improve knowledge of an under-served, under researched and under valued population, both geographically and demographically. The commitment to co-design in the proposal should quite likely lead to a larger and possibly successful larger research grant. The methods are very appropriate for the proposed work.

It is very likely this work can be completed in the time allocated and the commitments of the project are reasonable

The budget is appropriate for the proposed work.

Potential Impact of the Research

I believe the proposed work could meaningfully improve health experience, health outcomes and improved community design for older adults.

The partnership is diverse and strong

The proposed work could very meaningfully contribute to the improvement of health care systems and practices that support patient-oriented research for older adults.

Seemingly quite likely that the proposed project will inform and/or result in a future funding application for a more comprehensive

**Review Type/Type d'évaluation:**	Committee Member 2/Membre de comité 2
**Name of Applicant/Nom du chercheur:**	Bath, Brenna Lea
**Application No./Numéro de demande:**	425009
**Agency/Agence:**	CIHR/IRSC
**Competition/Concours:**	2019-09-10 Catalyst Grant: Patient-Oriented Research/Subvention catalyseur : Recherche axée sur le patient
**Committee/Comité:**	Catalyst Grant : Patient-Oriented Research/Subvention catalyseur : Recherche axée sur le patient
**Title/Titre:**	Patient experiences of health care access challenges for back pain care across the rural-urban continuum

Assessment/Évaluation:

patient-oriented research project.

**Review Type/Type d'évaluation:**	Committee Member 2/Membre de comité 2
**Name of Applicant/Nom du chercheur:**	Bath, Brenna Lea
**Application No./Numéro de demande:**	425009
**Agency/Agence:**	CIHR/IRSC
**Competition/Concours:**	2019-09-10 Catalyst Grant: Patient-Oriented Research/Subvention catalyseur : Recherche axée sur le patient
**Committee/Comité:**	Catalyst Grant : Patient-Oriented Research/Subvention catalyseur : Recherche axée sur le patient
**Title/Titre:**	Patient experiences of health care access challenges for back pain care across the rural-urban continuum

Assessment/Évaluation:

**Review Type/Type d'évaluation:**	Committee Member 3/Membre de comité 3
**Name of Applicant/Nom du chercheur:**	Bath, Brenna Lea
**Application No./Numéro de demande:**	425009
**Agency/Agence:**	CIHR/IRSC
**Competition/Concours:**	2019-09-10 Catalyst Grant: Patient-Oriented Research/Subvention catalyseur : Recherche axée sur le patient
**Committee/Comité:**	Catalyst Grant : Patient-Oriented Research/Subvention catalyseur : Recherche axée sur le patient
**Title/Titre:**	Patient experiences of health care access challenges for back pain care across the rural-urban continuum

Assessment/Évaluation:

Patient experiences of health care access challenges for back pain care across the rural-urban continuum

Research Approach:

This is original work looking at the barriers to back pain care in both Indigenous and non-Indigenous populations in Saskatchewan. This is a mixed model approach to the various hypotheses. Their goal is to lead to a patient-driven development of a comprehensive set of measures of CBP care access that are most meaningful to rural, remote and urban Indigenous and non-Indigenous patients.

Then the measures will be used in future studies to evaluate current and future community-based CBP interventions and cross provincial/ national comparisons, ultimately leading to enhanced access to more patient-centred care for CBP.

There are several phases to the study and a developed Knowledge dissemination plan.

Phase 1 = Qualitative interviews to develop survey
Phase 2 = Deployment of telephone survey to 383 individuals in Saskatchewan.
Phase 3 = An interactive end-of-project knowledge translation event.

Feasibility:

Team seems feasible – the random cold calling seems tough in today's environment

Solid team – likely doable in the year timeframe

Impact of the Research:

Clearly the strength lies in the mixed populations and the attempt to span several cross cultural themes. Clearly patient oriented and a good knowledge dissemination plan with appropriate timelines outlined. Fundable.

**Review Type/Type d'évaluation:**	Committee Member 4/Membre de comité 4
**Name of Applicant/Nom du chercheur:**	Bath, Brenna Lea
**Application No./Numéro de demande:**	425009
**Agency/Agence:**	CIHR/IRSC
**Competition/Concours:**	2019-09-10 Catalyst Grant: Patient-Oriented Research/Subvention catalyseur : Recherche axée sur le patient
**Committee/Comité:**	Catalyst Grant : Patient-Oriented Research/Subvention catalyseur : Recherche axée sur le patient
**Title/Titre:**	Patient experiences of health care access challenges for back pain care across the rural-urban continuum

Assessment/Évaluation:

Research Approach

This study aims to identify barriers to health care access for chronic back pain of remote, rural and Indigenous dwellers. This will lead according to the applicants to improved access to patient-centered care for back pain across geographical situations and cultural backgrounds. To do this, the applicants propose to explore lived experiences of back pain patients, co-create indicators of back pain care access and effectiveness, and identify programs or policy changes that could be implemented. These objectives will be achieved using a mixed methods design. The project focuses on gathering the opinion (qualitatively and quantitatively) of patients from different profiles as well as health care providers in order to gather necessary information for future patient-oriented research projects.

There is a vast body of knowledge on systemic barriers to health care access in Canada, not only for back pain but for all types of chronic pain. It was difficult to fully appreciate the novelty and need for this project given what is already known. The Canadian Task Force on Chronic Pain was launched last year and has a 3-year mandate on increasing access to chronic pain treatment. They have produced their first report this June of access to chronic pain treatment across the country and across different subpopulations. As such, it is difficult to see what more this study will bring to the table in the context of an important national endeavor already going on. At the minimum how are the applicants planning on informing and being informed by the work of this national task force?

The research idea stems from prior work from the research team and identified by patients. Patients are included in the research team and patient perspective will be prioritized during open dialogue during team meetings. They will be involved in conducting some of the interviews, interpret outcomes and disseminate knowledge. How this will be realized concretely however is unclear. In particular 4 meetings throughout the project does not guarantee complete involvement of patient partners.

Sex- and Gender-Based Analysis Plus (SGBA+) is discussed but not fully integrated within the proposal. It is unknown whether selection of participants will ensure adequate representation of sex and gender or how it will be taken into account in the analysis or dissemination. There are no concrete examples given as to how dissemination messages or modalities for example will be targeted to the different needs of men and women.

Feasibility

The team is composed of researchers with significant experience in the proposed subject. There are also 3 patient partners involved in the study who suffer from chronic back pain. Different policy makers and stakeholders have signed letters of collaboration.

The timeline is realistic and reasonable. The project is well circumscribed and delineated.

Budget seems reasonable and includes in-kind contributions.

Potential Impact of the Research

**Review Type/Type d'évaluation:**	Committee Member 4/Membre de comité 4
**Name of Applicant/Nom du chercheur:**	Bath, Brenna Lea
**Application No./Numéro de demande:**	425009
**Agency/Agence:**	CIHR/IRSC
**Competition/Concours:**	2019-09-10 Catalyst Grant: Patient-Oriented Research/Subvention catalyseur : Recherche axée sur le patient
**Committee/Comité:**	Catalyst Grant : Patient-Oriented Research/Subvention catalyseur : Recherche axée sur le patient
**Title/Titre:**	Patient experiences of health care access challenges for back pain care across the rural-urban continuum

Assessment/Évaluation:

In line with the reviewer's previous comment on the ongoing Canadian Task Force, it is unclear what this research will further contribute to the accumulating knowledge on treatment barriers. It would have been important to also assess how these barriers could be optimally addressed rather than identifying them (we already have a good idea of the barriers).
The idea of generating indicators is interesting and pertinent for guiding future research and be able to evaluate the effectiveness of interventions geared at reducing barrier access.
